# An Overground Robotic Gait Training Program for People With Multiple Sclerosis: A Protocol for a Randomized Clinical Trial

**DOI:** 10.3389/fmed.2020.00238

**Published:** 2020-06-09

**Authors:** Rakel Berriozabalgoitia, Begoña Sanz, Ana Belén Fraile-Bermúdez, Erika Otxoa, Izaskun Yeregui, Iraia Bidaurrazaga-Letona, Iratxe Duñabeitia, Alfredo Antigüedad, Maria Domercq, Jon Irazusta, Ana Rodriguez-Larrad

**Affiliations:** ^1^ADEMBI, Multiple Sclerosis Association of Bizkaia, Bilbao, Spain; ^2^Department of Physiology, Faculty of Medicine and Nursing, University of the Basque Country (UPV/EHU), Leioa, Spain; ^3^Department of Nursing I, Faculty of Medicine and Nursery, University of the Basque Country (UPV/EHU), Leioa, Spain; ^4^Biocruces-Bizkaia Research Institute, Department of Neurology, Cruces University Hospital, Osakidetza Basque Health Service, Barakaldo, Spain; ^5^Department of Neurosciences, University of the Basque Country, Achucarro Basque Center for Neuroscience-UPV/EHU, Centro de Investigación Biomédica en Red de Enfermedades Neurodegenerativas (CIBERNED), Leioa, Spain

**Keywords:** exoskeleton, gait speed, multiple sclerosis, rehabilitation, inflammation biomarkers, quality of life

## Abstract

Maintaining the ability to walk is one of the significant challenges in people with multiple sclerosis (MS) for keeping a good quality of life as the disease and the aging process progresses. Overground robotic (OR) wearable exoskeletons are promising tools for gait rehabilitation, but currently there is no evidence of their clinical effects on patients with MS. The present study aims to determine the effects of an OR intervention in people with MS and moderate to severe walking disabilities and ascertain if benefits are maintained over a follow-up period of 3 months. This randomized controlled trial will include 36 participants with MS. Inclusion criteria are: older than 18 years, definitive diagnosis of MS, 4.5–7 points on the EDSS (Expanded Disability Status Scale), and needing one or two canes or crutches for walking outdoors. Subjects in the control group will receive conventional physiotherapy sessions at ADEMBI (Asociación de Esclerosis Múltiple de Bizkaia) provided to control spasticity, maintain articular range and exercise balance. Subjects in the intervention group will receive the same physiotherapy but also participate in a progressive OR gait training program assisted by the Ekso^TM^ exoskeleton. The program consists of twice a week individually supervised sessions in two setting modalities: *PreGait* and *ProStepPlus*. The training parameters (duration, speed, cadence, length of steps) will be set during the first session and the progression and intensity of the intervention will be adapted to the tolerance of each participant. The primary outcome of this study is gait speed. Secondary outcomes will include physical and cognitive performance tests, clinical, fatigue and quality of life assessments, and changes in the plasma levels of inflammatory cytokines. The present trial is the first analyzing the effectiveness of an OR intervention for gait training in patients with MS. It will help clarify the applicability of robotic technologies to clinical practice, extending the functionality and quality of life of people with MS to face a successful aging process. (ACTRN12619000014156; https://anzctr.org.au/Trial/Registration/TrialReview.aspx?id=376548).

## Introduction

Multiple sclerosis (MS) is a patchy, inflammatory, autoimmune, and demyelination disorder of the central nervous system characterized by the accumulation of progressive neurological impairment (1). MS presents with a wide range of neurological symptoms related to the varying degree and location of axonal and neuronal damage. The disease can follow a variety of clinical courses and is unpredictable in terms of prognosis. Reduced mobility and gait dysfunction are the most noticeable signs of MS; up to 85% of people with MS report difficulty in walking (2, 3) after 10 years of disease onset. In addition, over 60% of people with MS experience some degree of cognitive impairment (4) that correlates with peripheral changes in pro-inflammatory cytokines such as interleukin 1β (IL-1β) and tumor necrosis factor α (5). Fatigue and postural control impairment also appear ubiquitous over the course of MS (6). Increasing evidence suggests that impaired mobility and symptoms such as fatigue are important factors contributing to the observed reduction in quality of life (7) and, in some cases, increased costs (8) associated with MS. In fact, from mid-life and on, MS disease progression can negatively impact employment and participation in everyday activities (9).

An observational study (10) conducted in Canada reported a rise in the prevalence of MS from 1984 - 1997 specifically among people in older age groups. This occurred with minimal changes in the incidence, possibly indicating that patients with MS were living longer. The same trend was also observed in other epidemiological studies conducted in Europe (11–13). As they age, people with MS are at increased risk for developing comorbidities (14–16), at a time when they are often experiencing a progression in their disability (17). Older adults with MS experience limitations in daily-life activities, report low health-related quality of life status, and express concerns about the continuous loss of function and mobility with aging (18–21). People aging with MS also report lower freedom perception relative to that of their cohort peers and fear that they may become a burden on family members (22). In summary, people living with MS are confronted with the challenge of dealing with the functional deterioration associated with disease progression while simultaneously dealing with age-related changes. The resulting decline in functional capacity frequently occurs at a very young age, leading to approach it as a premature aging process.

People with MS perceive gait as one of the most valuable physical functions (23), and consequently regard impaired walking ability as a major physical problem (24). Accordingly, it is of utmost importance to preserve MS patient's capacity to locomote as long as possible to allow for successful aging. Rehabilitation strategies for MS gait dysfunction have experienced significant improvement in recent years and have incorporated new technologies (25). For example, robot-assisted gait training (RAGT), which has shown to be feasible in patients with spinal cord injuries (26) and stroke (27), has also been evaluated in MS (28). RAGT consists on a training modality in which the patient's body weight is supported by a harness while walking on a treadmill with the help of a robotic-driven gait orthosis (29). Morone et al. (30) have identified the following characteristics of RAGT devices: i) capable of mobility with different levels of autonomy, ii) could be classified as either “exoskeletons” (when the movement of specific joints is controlled [i.e. hip, knee or ankle joint]) or “end-effect robots” (the device is at the end of the limb, [i.e. the feet are placed on a footplate]) and iii) could be classified as static (when the patient remains in a fixed environment) or dynamic (if the device enables to change the location). RAGT allows therapists to facilitate the task-oriented practice of walking (31), which potentially leads to learning-dependent neuroplasticity (32). RAGT allows intensive gait training without highly demanding therapists and patients, particularly when treating patients with severe gait impairments.

However, RAGT does not appear to be superior to intensive overground walking training in people with MS. In a recently published randomized controlled study of seventy-two patients affected by severe disability (EDSS 6-7), the effects of 4 weeks of RAGT intervention was compared with conventional walking training in terms of gait speed (33). Both therapies were found to significantly improve gait speed with respect to baseline assessment, but the study failed to demonstrate group by time interactions. Similar improvements were observed for most of the secondary outcomes: walking, endurance, balance, fatigue, and quality of life. However, the improvements obtained during the interventions were lost within a few months of program cessation. Additional studies evaluating RAGT interventions in people with MS and severe walking disabilities (28, 34–37) have yielded similar results. Thus, there is a clear need to understand how best to create stable adaptations that can support and improve the long-term mobility of patients with MS.

The overground robotic exoskeleton (OR) is a relatively new advanced technical device being incorporated into the field of gait rehabilitation. ORs are wearable powered exoskeletons that allow individuals to walk on hard and flat surfaces in a real-world setting. They require a patient's active participation and provide greater motor control stimulation and enriched multisensory information (visual, proprioceptive, tactile, and vestibular) than the RAGT systems (38). Preliminary findings suggest that OR-assisted gait training may be more beneficial than conventional walking training in post-stroke patients (39). A recent study of forty patients with hemiparesis due to stroke compared the effectiveness of OR and conventional gait training in the chronic recovery phase (40). OR gait training resulted in a significant improvement in gait speed, coordination of muscle activations, and frontoparietal effective connectivity. Additionally, recent systematic reviews indicate that OR is a feasible treatment for patients with spinal cord injury (SCI), allowing safe ambulation in real-world environments (41, 42). However, the effectiveness of OR gait training interventions for improving mobility in people with MS has not yet been studied.

Therefore, this study aims to determine the effects of the addition of an OR intervention to the conventional rehabilitation program on gait speed in people with MS and moderate to severe walking disabilities. As a secondary aim, the study will determine the effects of the intervention from a multidimensional perspective, taking into account physical, cognitive and clinical parameters, fatigue and health-related quality of life of the participants. Additionally, as MS is accompanied or mediated by neuro-inflammation, training interventions may stimulate an anti-inflammatory response contributing to the positive effects of exercise in patients with MS (43, 44). Thus, the influence of the intervention on peripheral inflammatory cytokines will also be analyzed. Finally, this study aims to ascertain if the effects of the intervention are maintained over a follow-up period of 3 months.

## Materials and Methods

### Study Design and Participants

**Figure 1:**
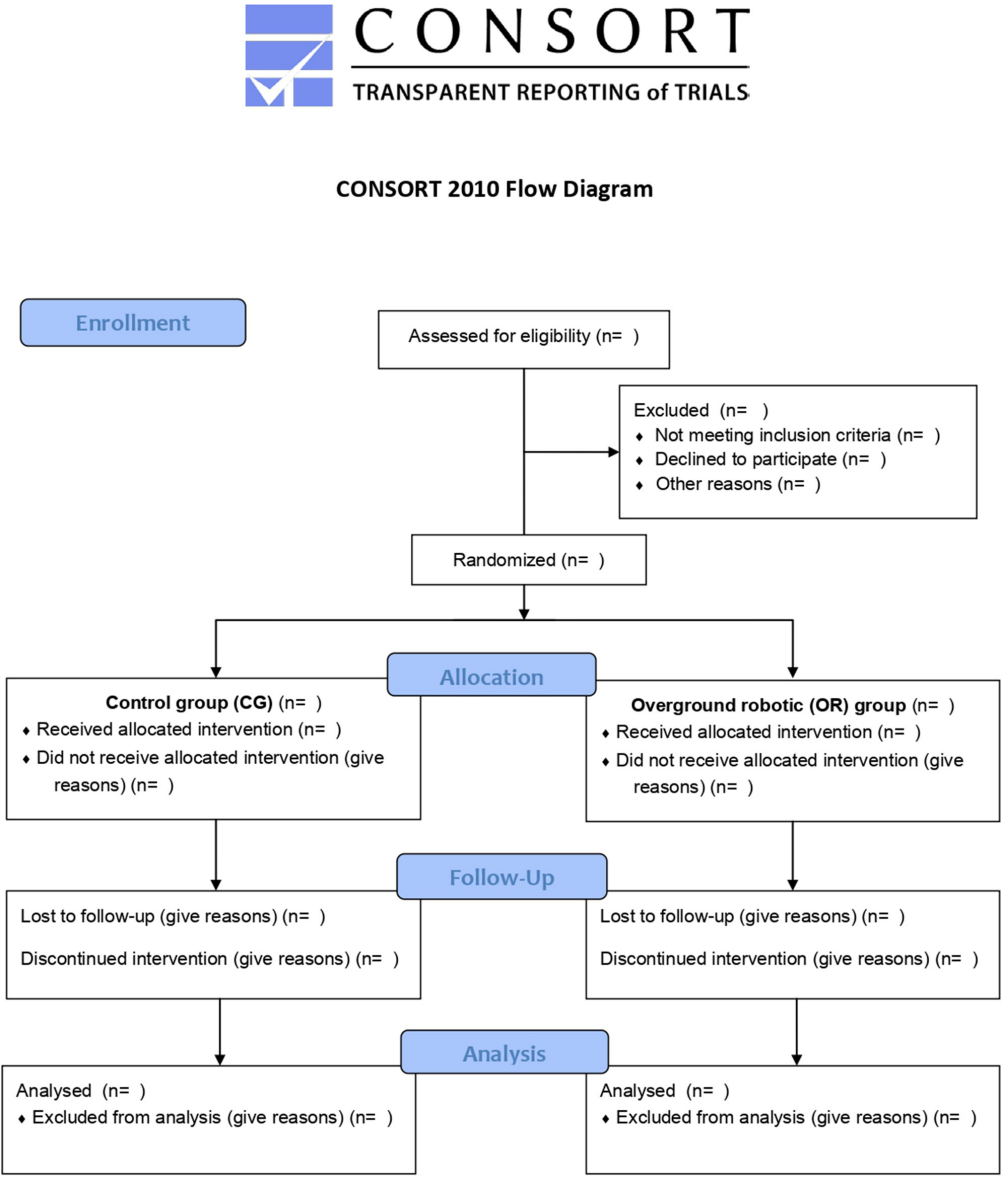
Flow diagram of the study.

This is a prospective parallel-assignment, single-blinded, randomized controlled study. Participants will be randomly assigned to one of the following groups: the control group (CG) or the OR group. The study will take place at the Asociación de Esclerosis Múltiple de Bizkaia (ADEMBI) between January 2019 and December 2019. The assessments will be conducted by blinded research staff at baseline (T1) and after 3 months of training (T2). After a 3-month follow-up period, a final assessment will be performed at 6 months (T3). The study has been designed and results will be reported according to the CONSORT Statement extension for trials of non-pharmacological interventions and pragmatic intervention trials (Figure 1

). This study was approved by the Ethics Committee of Clinical Research of Euskadi (CEIm) (Code PS2018017) and by the Ethics Committee for Research with Biological Agents of the University of the Basque Country (CEIAB) (Code M30/2018/073).

### Inclusion and Exclusion Criteria, Recruitment, and Randomization

Subjects will be considered eligible for the study if they fulfill all of the following criteria: (a) aged ≥ 18 years, (b) diagnosis of MS according to McDonald criteria (45), (c) moderate to severe gait impairments defined by an Expanded Disability Status Scale (EDSS) score ranging from 4.5 to 7 (46), and (d) the need for one or two canes or crutches for walking outdoors.

Participants will be excluded if they (a) have a neurological pathology in addition to MS, (b) have some musculoskeletal disorder that could limit the extension of the hip and knee or the plantar flexion of the ankle, (c) have suffered an outbreak of MS during the 3 months prior to the start of the study, (d) are under non-stable pharmacological treatment or with treatments expected to be modified during the study, or (e) have received treatment with botulinum toxin in the 12 weeks prior to the beginning of the study.

Recruitment will be done at ADEMBI. Informative meetings will be organized to provide detailed oral and written information about the study, including objectives, studied variables, and methodology, to both potential participants and their families. Participants will sign, indicating informed consent, after fully understanding the study. Using a coin-tossing sequence generation, participants will be randomly allocated at a 1:1 ratio to either the control group (CG) or the intervention group (OR). The assignment will be conveyed via sealed opaque envelopes.

### Control Group Intervention (CG)

Subjects in the CG will participate in the routine physiotherapy sessions provided to all patients with MS at ADEMBI. ADEMBI is a reference MS center for providing expertise and specialized rehabilitation to people with MS, where all the participants receive individualized assistance throughout the year following the latest recommendations and clinical guidelines for managing the disease. The duration of each session is one hour; in these sessions, conventional methods of physiotherapy are provided to control spasticity, maintain articular range and exercise balance.

### Overground Robotic Gait Training Group (OR)

Subjects in the intervention group will receive the same physiotherapy treatments scheduled for the control group and will participate in 3 months of individualized and progressive gait training assisted by an OR (Ekso^TM^, Ekso Bionics, USA). Ekso^TM^ is a powered, wearable exoskeleton with actuated hips and knees that provides assistance to lower limb segments during movement. The gait training program's technical content is based on a literature review, author's expertise, and field experience (Table 1). It has been designed taking into account the multi-modal training approach, as has been recommended by expert physical therapists on the rehabilitation of patients with MS (47), to allow the “far transfer effect”. This effect refers to the occurrence of transferring the improved performance from a specific function to different untrained functional domains (48). Interventions will be individually supervised by experienced physiotherapists properly trained in Ekso^TM^ driving and will consist of twice weekly sessions assisted by the wearable exoskeleton in two modalities: 1) *PreGait*: to exercise static balance and weight shifts at the beginning of each session; 2) *ProStepPlus:* to gait train, with steps triggered by the user's lateral weight shift. Subjects will wear their own shoes and orthotics during the training sessions. The parameters (duration, speed, cadence, length of the steps, robotic assistance, etc.) of the intervention will be adapted to each participant, and the duration of the training sessions progressively increased as participants increase their tolerance, aiming to achieve 20-40 minutes of OR training (including the exoskeleton donning and doffing) by the end of the 3 month training period. The session will end with 10 minutes of cryotherapy on knee extensors and ankle plantar flexors. There will be at least 48 hours between training sessions, and goals will be adapted in response to illness, injury, or physical symptoms.

**Table 1:** Technical content of the OR gait training program.

***PreGait***	2–3 exercises of static weight shift in progressive difficulty to identify:		
	(a) Symmetric distribution of weight		
	(b) A given lateral weight displacement point		
	**1st Month**	**2nd Month**	**3rd Month**
	Patients assisted by acoustic stimuli	Reduction of external stimuli and lateral weight displacement point	Lateral weight displacement and step on the site
*ProStepPlus*	Gait training sessions of progressive length and difficulty according to patient tolerance (maximum 40 min/session)		
	**1st Month**	**2nd Month**	**3rd Month**
	Patients assisted by acoustic stimuli when reaching the lateral point to trigger the step	Reduction of external stimuli and lateral weight displacement point trigger	Increase the duration and speed of the sessions targeting a functional gait
	Ekso^TM^ bilateral full adaptive assistance	Ekso^TM^ bilateral full adaptive assistance	Ekso^TM^ bilateral full adaptive assistance
Cryotherapy	10 min on knee extensors and ankle plantar flexors in a supine position.		

### Primary Outcome: Gait Speed

The assessments will be performed at ADEMBI by blinded research staff at baseline (T1), 3 months (T2), and 6 months (T3). Participants will walk through a 10-meters corridor at their own habitual speed. The time spent in performing the test will be measured through photoelectric cells (Polifemo, Microgate, Italy), and afterwards, gait speed calculated Participants will be allowed to wear their own footwear, orthoses, and prescribed usual assistive device (crutches, canes). This test will be performed as the first assessment to avoid possible muscle fatigue.

### Secondary Outcomes

Secondary outcomes will include physical and cognitive performance tests (Table 2), and clinical, fatigue and quality of life assessments (Table 3), and the measurement of circulating inflammatory cytokines.

**Table 2:** Physical and cognitive assessment tests and questionnaires.

**Test (references)**	**Functions/Parameters**	**Description**
Handgrip strength test (Jamar dynamometer) (46)	Dominant hand grip strength	Squeeze the dynamometer with maximum isometric effort for about 5 s
Timed Up and Go test (47)	Dynamic balance	Get up from a chair without upper-limb assistance, walk 3 m at a normal pace, turn and sit back in the chair
Short Physical Performance Battery (SPPB) (48)	Lower extremity function: static balance, gait speed, and getting in and out of a chair	Side-by-side, semi-tandem, and tandem stands (10 s); 4 m walk test at comfortable speed; and 5 quick sit to stands from a chair without upper-limb assistance
Berg balance test (49)	Postural stability	Performance of 14 functional tasks
Yale Physical Activity Scale (50)	Physical activity	Type, amount, pattern, and perceived difficulties of performed physical activities throughout daily living
Montreal Cognitive Assessment (MoCA) (51)	Cognitive dysfunction	Covered domains: attention and concentration, executive functions, memory, language, visuoconstructional skills, conceptual thinking, calculations, orientation

**Table 3:** Muscle spasticity, fatigue, and health related quality of life assessment tests and questionnaires.

**Test (References)**	**Functions/Parameters**	**Description**
Modified Ashworth Scale (MAS) (52)	Spasticity	Graduation of the resistance encountered during passively stretching flexor and extensor muscle groups of bilateral hip, knee, and ankle
Modified Fatigue Impact Scale (MFIS) (53)	Physical and mental fatigue	21-item scale measuring perceived fatigue over the last four weeks
EuroQol-5 (54)	Health related quality of life	Covered domains: mobility, self-care, usual activities, pain/discomfort, and anxiety/depression

Participants will self-report their age, gender, marital status, employment situation, and Barthel index (49). Physical and cognitive examination will include the following (Table 2): the handgrip strength test (50) (Jamar dynamometer), Timed Up and Go test (51), Short Physical Performance Battery test (52) (SPPB), Berg balance test (53), Yale Physical Activity Survey (54) (YPAS), and Montreal Cognitive Assessment (55) (MoCA). Clinical information will be determined by the Modified Ashworth Scale (56) (MAS) and the number of outbreaks and falls during the studied period. Fatigue will be assessed by the Modified Fatigue Impact Scale (MFIS) (57) and health related quality of life by the EuroQol-5 [(18); Table 3].

Plasma levels of pro-inflammatory (Interferon γ, Interleukin-12, Tumor necrosis factor-α, Interleukin-17, and Interleukin-6) and anti-inflammatory (Interleukin-6, Interleukin-4, Interleukin-13, Interleukin-10 and Tumor growth factor-β) cytokines will be assessed using Simoa HD-1 technology. Assays will be performed according to the manufacturers' instructions. Briefly, a two-step assay procedure will be utilized. Standards will be prepared with calibrator diluent according to lot-specific concentrations provided by the manufacturer. Samples will be prepared as directed using a sample diluent off-line.

### Safety Assessments

All co-existing diseases or conditions related to the intervention will be treated according to prevailing medical practice. They will be reported as adverse events. In cases where the functionality of a participant decreases due to an adverse consequence (e.g., skin injuries, falls, etc.) the program will be individualized and adapted for that person upon her/his return.

### Sample Size Calculation

Sample size has been calculated to detect a clinically meaningful change in gait speed: accepting an alpha risk of 0.05 and a beta risk of 0.20 in a bilateral contrast, 32 subjects are required to detect a difference equal to or greater than 0.1 m/sec speed in the 10 meters timed walking test (SD = 0.16). Sample size has been increased an additional 12% to account for losses during follow-up. The resulting sample size is 36 subjects, therefore 18 individuals per group (intervention and control group).

### Statistical Considerations

Data analysis will be performed using the IBM SPSS Statistics 24 statistical software package (SPSS, Inc., Chicago, IL). First, all data will be checked for normality of distribution using the Shapiro-Wilk test. Results will be expressed as means (with standard deviations) for continuous and normally distributed variables and as medians (with interquartile ranges) when normality of data cannot be assumed. Categorical variables will be described in frequency counts and percentages. Tests for baseline comparisons will be selected based on the nature and distribution of the data: Student's-t test for continuous and normally distributed variables, Mann-Whitney test for non-normally distributed continuous variables, and Chi-squared test for categorical variables. To test the effects of training interventions, mixed-designed ANCOVA-s or the Friedman test, including baseline measurements as covariates, will be performed for all studied variables. In cases where a significant F-value is found, LSD *post hoc* procedures will be performed for pair-wise comparisons. The level of statistical significance will be set at *p* < 0.05 for all computations.

### Trial Status

The trial is currently ongoing with participants taking part in the OR intervention. The study is expected to finish by December 2019.

## Discussion

To the knowledge of the authors, the current trial is the first analyzing the effects of an OR intervention for gait training in people affected by MS. In particular, this study may provide valuable information on the feasibility and effectiveness of an intervention designed specifically for these particular patients with moderate to severe gait impairment. Besides, the effects of the intervention will be assessed from a multidimensional perspective, taking into account physical, cognitive and clinical parameters, fatigue and health-related quality of life of the participants. It will also address how the intervention affects inflammation by measuring circulating inflammatory cytokines. Therefore, the results will help better the understanding of the role of ORs in expanding the time of functionality and quality of life of these patients and allow for healthy aging.

This study could also clarify the applicability of robotic technologies in clinical practice, identifying the optimal duration and frequency of the sessions that patients with MS can tolerate and determining the type of exercises, intensities, and gait kinematics that they can accept (duration, speed, cadence, length of steps, etc.). Additionally, the proposed study will assess if the effects of an OR intervention can be maintained over a follow-up period of 3 months. Since the benefits of other therapies, such as RAGT (33), have proven to fade with time, it is imperative to identify interventions that can provide more lasting benefits.

There are few evidence-based therapies using robotic technologies for people with MS, despite the growing interest in robotics in clinical practice. Furthermore, there is, of yet, no clear guidance regarding the specifications of such therapy for this population, and available studies rarely offer detailed information on the design of the assessed interventions, confounding replication. Moreover, taking into account the heterogeneity of these patients' characteristics, there is an urgent need for establishing tailored robotic-based therapies for MS-particular impairments. Future research should focus not on the global concept of robotic-based therapies, but on which specific intervention is the most cost-efficient for the characteristics and therapeutic objectives of a determined population. From this point of view, the results of the present study would contribute to the development of guidelines that help clinicians and policymakers make evidence-based decisions that lead to the optimization of health care resources.

Methodological strengths of the present study include its randomized controlled design and a clear definition of participants' characteristics in terms of gait impairment (the need for one or two canes or crutches for walking outdoors). In addition, the assessed intervention will include exhaustive practical information regarding implementation, such as training frequency, OR's setting modalities, duration, intensity, individualization, progression, and resting periods. The goal is to provide professionals working in the field of neurorehabilitation with an easy and straightforward description of how to implement the proposed intervention.

Possible limitations are also recognized. The sample size calculation might not be enough for detecting effects on the secondary outcomes, necessitating further studies to verify how the intervention affects physical, cognitive, and clinical parameters, fatigue and quality of life of the participants, and the levels of circulating inflammatory cytokines.

## Ethics Statement

The study has been approved by the Ethics Committee of Clinical Research of Euskadi (CEIm) (Code PS2018017) and by the Ethics Committee for Research with Biological Agents of the University of the Basque Country (CEIAB) (Code M30/2018/073). All participants will provide written informed consent based on documents approved by the Ethics Committee of Clinical Research of Euskadi Review Board. In addition, the study will be conducted in accordance with Good Clinical Practice, applicable local regulatory requirements, and the guiding principles of the Declaration of Helsinki.

## Author Contributions

AR-L, JI, EO, AA, and MD conceived and designed the study. BS, AF-B, IB-L, AA, and ID will be responsible for the collection of the data. RB, EO, and IY will recruit participants and perform the intervention. AR-L, JI, IB-L, and ID will analyze and interpret the data. AR-L, JI, IB-L, BS, and MD drafted this design paper. All authors read and approved the final manuscript.

## Conflict of Interest

The authors declare that the research was conducted in the absence of any commercial or financial relationships that could be construed as a potential conflict of interest.

## Abbreviations

MS: multiple sclerosis
RAGT: robot-assisted gait training
EDSS: Expanded Disability Status Scale
OR: overground robotic
ADEMBI: Asociación de Esclerosis Múltiple de Bizkaia.
